# Management of acute proliferative diabetic retinopathy related complications during the first COVID-19 wave

**DOI:** 10.1186/s12886-022-02349-3

**Published:** 2022-03-12

**Authors:** Niku Dhillon, Cynthia Santiago

**Affiliations:** Department of Ophthalmology, Aberdeen Royal Infirmary, Aberdeen, AB25 2ZN Scotland

**Keywords:** Proliferative diabetic retinopathy, Vitreous haemorrhage, Lockdown, COVID-19

## Abstract

**Background:**

Routine hospital eye services (HES) across the National health service (NHS), and diabetic eye screening (DES) in Scotland were paused during the COVID-19 lockdown in March 2020. Alternate pathways for managing acute ophthalmic pathology were devised in NHS Grampian covering the North-East of Scotland. Emergency eye treatment centres (EETC) manned by community optometrists were set up to treat and triage referrals to HES.

**Methods:**

Retrospective study analysing consecutive patients referred to a tertiary eye centre (Aberdeen Royal Infirmary) with proliferative diabetic retinopathy (PDR) related complications between March and August 2020. General demographical data, diabetic history, visual acuity, ocular complication, type of management, time to follow-up, and any appointment cancellations were extracted for analysis.

**Results:**

Fifty two eyes of 46 patients with PDR related complications were identified. HES appointment had been delayed or cancelled in 22 patients (48%) due to COVID-19. Mean age was 54.5 years (±15.1), 21 (46%) were female, 21 (46%) had type 1 diabetes; mean HbA1c was 78 mmol/l (±18.7). Vision ranged from 6/6 to perception of light. 36 (78%) patients had unilateral vitreous haemorrhage (VH), 6 (13%) bilateral, 2 (4%) tractional retinal detachments and 3 (6.5%) had neovascular glaucoma.

Of 48 acute PDR presentations, 18 (38%) were given anti-VEGF within 72 h and two (4%) had PRP the same day. 16 (33%) were rebooked into the laser clinic, 13 (27%) referred for urgent surgical review, and 17 (35%) advised observation and review in clinic. After a median follow-up of 6 months, 12 eyes (23%) of 11 patients progressed to have vitrectomy.

**Conclusion:**

Despite lockdown, hospital appointment cancellations and recommended footfall reduction limiting capacity due to COVID-19, patients reaching out with PDR complications were promptly referred to HES and appropriate treatments carried out with COVID-19 precautions as recommended.

## Introduction

Government COVID restrictions in March 2020 forced hospital departments to cancel all non-urgent appointments and re-design ophthalmic services [1]. Emergency optometry and hospital eye services (HES) remained open to prioritise sight threatening presentations. Diabetic eye screening (DES) in Scotland was also paused during the period of lockdown [2].

A new pathway for acute ophthalmic pathology was created: patients underwent initial assessment by one of six emergency optometry practices across NHS Grampian before referral onto HES [3]. The ophthalmology department in Aberdeen royal infirmary caters to a population of 600,000 in the north east of Scotland including Aberdeen city, Aberdeenshire, Moray and Shetland islands [4].

Patients with proliferative diabetic retinopathy (PDR) often remain asymptomatic and are picked up in screening. Complications of PDR are sight threatening and include vitreous haemorrhage, tractional retinal detachment and neovascular glaucoma, all of which require urgent treatment. Mainstay of risk reduction is the early detection of retinal neovascularisation (PDR) achieved through regular and as recommended diabetic eye screening through DES service and optimisation of systemic risk factors including glycaemic and blood pressure control [5].

Patients with PDR related complications usually present with sudden onset floaters and/or loss of vision. Painful loss of vision in the context of PDR, usually indicates end stage disease complicated by neovascular glaucoma and poor visual prognosis. All symptomatic patients require urgent ophthalmic assessment in the form of visual acuity and intraocular pressure (IOP) measurements with anterior segment and dilated fundus examination. In the presence of vitreous haemorrhage and poor fundus visualisation, B-scan ultrasonography is carried out to rule out retinal detachment.

Once PDR is identified, prompt treatment with laser panretinal photocoagulation (PRP) is standard practice to reduce the ischaemic effects of vascular endothelial growth factor (VEGF) [6] and avoid irreversible vision loss. Intravitreal injections of anti-VEGF medication are an alternative treatment modality [7]. Due to the invasive nature with associated risks [8] and the need for repeated treatments, antivegf injections as primary treatment without PRP are recommended for patients who can attend regular followups [9]. Vitrectomy is indicated for non-resolving vitreous haemorrhage, significant retinal traction and detachment.

Without prompt management of PDR, patients are at high risk of poor long-term visual outcomes. The impact of COVID-19 restrictions on diabetic retinopathy have been explored: a significantly lower number of intravitreal anti-VEGF injections were reported to be administered during the height of the pandemic [10] by Ahmed et al. while Stone et al. reported that only one third of scheduled patients were seen and treated [11]. Karempela et al. [12] also reported significantly fewer visits for retinal laser during this period and delays in care that led to poorer visual outcomes [13]. A large cross-sectional study in India observed an increase in patients presenting with proliferative diabetic retinopathy and significant visual impairment over the lockdown period compared to the previous year [14]. Chatziralli et al. described how restricted access to hospital follow-up and treatment led to poorer visual outcomes and progression in PDR [15]. Patient records including those with diabetic retinopathy under HES care were reviewed in many centres and those categorised as high risk for visual loss were offered priority hospital appointments [16], and a similar approach was followed in our centre. Despite such efforts, BBC news reported that up to 50% of all ophthalmic patients were not attending their most urgent appointments and observed a 30% non-attendance rate for procedures [17].

The existing literature does not describe how patients with PDR presented or how they were managed acutely upon entering the hospital eye service over the first COVID-19 wave. Here we aim to describe the complications of PDR in patients who presented to the emergency eye service during the COVID-19 pandemic.

## Methods

This observational retrospective study analysed all patient records of those who presented to Aberdeen Royal Infirmary, Aberdeen, United Kingdom with a complication attributed to PDR between 23rd March 2020 and 31st August 2020 inclusive. Diabetic patients who presented with other ocular complaints not related to PDR, including haemorrhagic posterior vitreous detachment, were excluded. Follow-up of included patients continued for a minimum of 6 months, until 23rd March 2021.

During lockdown, patients were able to present acutely to the hospital eye service (HES) upon referral from either acute optometry, general practice, other hospital-based department or self-presentation. Referrals were made to HES by telephone or email via a central triage hub, the clinical decision unit (CDU). All email referrals received through the CDU email address and all telephone calls were audited during the period of COVID lockdown. Patients seen in the ophthalmic acute casualty service are entered into an Eye Health database and records can be accessed for audit purposes. The email address, casualty database and telephone data were searched for patients who presented with PDR complications.

Data extracted included general demographics, past ocular history, diabetic type, HbA1c, status of insulin dependence, vision at presentation, intraocular pressure (IOP), anterior and dilated fundus slit lamp examination, diagnosis, management, time to follow-up, type of follow-up and any appointments cancelled by the department.

The patient’s postcode was used to determine socioeconomic status by using the publicly accessible tool, the Scottish Index of Multiple Deprivation (IMD) 2020 [18]. The IMD is a standardised index used to identify places where people experience disadvantage across multiple domains. It is constructed from seven domains, income, employment, education, health, access to services, crime and housing. The lower the IMD score, the higher the deprivation in that area.

The primary aim was to report the presenting complication of PDR and effect on vision during the first wave of COVID 19. The secondary aims were to investigate the characteristics of high-risk eyes, how complications were managed and the effect of anti-VEGF and socioeconomic status on visual outcome.

No experimentation was performed in this study.

The study of this type did not require ethical permission or informed patient consent as it was viewed and registered as a service evaluation designed to address the changing environment, approved by the North of Scotland Ethics Committee. This study was conducted in accordance with the declaration of Helsinki and the UK’s Data Protection Act.

Demographic data are presented as mean with standard deviation (SD) and median with interquartile ranges (IQR) where appropriate. Statistical differences between patients that received intravitreal injection of anti-VEGF and those that did not were compared using the Student’s t-test and Mann-Whitney U test for parametric and non-parametric data, respectively. Pearson correlation coefficient was used to determine correlations between socioeconomic status and visual acuity.

## Results

Over the 5-month period 52 eyes of 46 patients presented acutely to hospital eye services (HES) with an acute complication of PDR. Two patients presented to HES acutely on two separate occasions, totalling 48 acute presentations to HES.

Twenty-one (46%) were female and 25 male (54%). Mean age was 54.5 years (±15.1; range 25–86). 21 (46%) had type one diabetes and 25 were type two (54%). 9 (36%) type two diabetics were tablet controlled, 6 (24%) required both tablet and insulin control, and 10 (40%) used insulin only. Mean glycosylated haemoglobin (HbA1c) was 78 mmol/mol (±18.7; range 41–140) (Table 1).

**Table 1:** Patient characteristics

Demographic	***N*** = 46
Age, years, mean ± SD [range]	54.5 ± 15.1 [25–86]
Sex, n (%)	
• Female	21 (46%)
• Male	25 (54%)
Socioeconomic status, decile ± SD [range] N.B. 1 being most deprived	6 ± 2.5 [1–10]
DM type, n (%)	
• Type 1	21 (46%)
• Type 2	25 (54%)
◦ Tablet	9 (36%)
◦ Tablet + insulin	6 (24%)
◦ Insulin	10 (40%)
HbA1c, mmol/mol, mean ± SD [range]	78 ± 18.7 [41–140]
Ocular status, n (%)	
• Vitrectomy	8 (15%)
• PRP	32 (62%)
• Anti-VEGF for PDR	17 (33%)
• Anti-VEGF for DMO	3 (6%)
• Anti-VEGF for PDR + DMO	2 (4%)
• Tx naïve	6 (12%)
Under hospital or community (DRS) care, n (%)	
• Hospital	29 (63%)
• DRS	17 (37%)
BCVA at presentation, logMAR, median (IQR) [range]	2.00 (0.4–2.30) [0.00–2.80]
BCVA at end of follow-up, logMAR, median (IQR) [range]	0.40 (0.20–1.00) [− 0.10–2.80]

*Abbreviations*: *BCVA* best corrected visual acuity, *DM* diabetes mellitus, *DRS* diabetic retinal screening, *HbA1c* glycated haemoglobin, *IQR* interquartile range, *n* number, *PDR* proliferative diabetic retinopathy, *PRP* panretinal photocoagulation, *SD* standard deviation, *VEGF* vascular endothelial growth factor

The median vision at presentation was 2.00 logMAR or counting fingers (IQR 0.40–2.30; range 0.00–2.80 or 6/6 - LP).

**Fig. 1:**
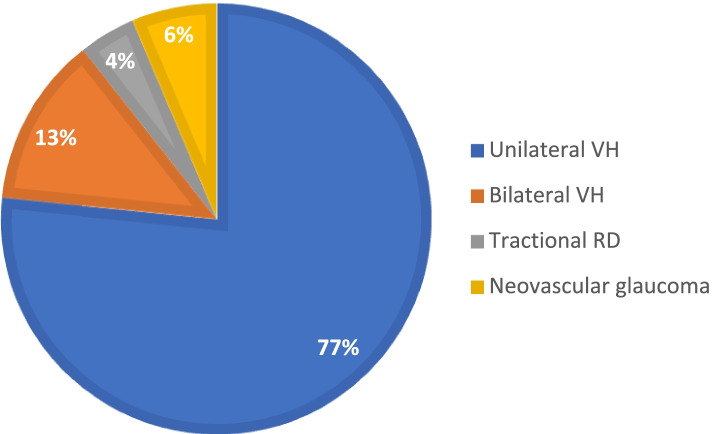
Type of proliferative diabetic retinopathy complication

Thirty-six patients presented with a unilateral vitreous haemorrhage; 6 patients presented with bilateral vitreous haemorrhages; two were diagnosed with a tractional retinal detachment; and three presented acutely with a new diagnosis of neovascular glaucoma (NVG) (Fig. 1).

One patient attended HES with a unilateral vitreous haemorrhage at the start of lockdown and subsequently presented with a vitreous haemorrhage in her other eye 4 months later. This was managed with an intravitreal injection of bevacizumab 2 mg at initial presentation in each eye, however, the patient subsequently failed to attend for follow-up.

Of those who presented with NVG, one was known to HES and had been previously treated with extensive bilateral PRP and anti-VEGF to the affected eye for diabetic vitreous haemorrhage. The scheduled follow-up appointment was delayed by COVID related hospital restrictions and subsequently presented with reduced vision and elevated pressure. The patient presented acutely twice with ocular pain secondary to elevated pressure, which was refractory to medical treatment. A multidisciplinary approach was taken with urgent referral onto specialist glaucoma and surgical care. They progressed onto further anti-VEGF injections, phacovitrectomy, cyclodiode laser and Ahmed valve implant. At 6 months post valve surgery pressure stabilised and vision improved from CF to 6/9.

The second case was a treatment naïve insulin dependent type 2 diabetic with poor glycaemic control. They were unknown to HES and had not engaged with diabetic screening for the past 6 years. Intraocular pressure was successfully managed with anti-VEGF injection and urgent PRP.

The third patient presented with NVG three months following uncomplicated cataract surgery. The patient was a tablet-controlled type 2 diabetic who was not known to HES and had not received previous treatment for diabetic retinopathy. The outcome of his last attendance at DES, 8 months prior, was mild non proliferative diabetic retinopathy with referrable maculopathy (R1M2) monitored in DES. Pressure stabilised with anti-VEGF injection, PRP and cyclodiode laser.

Eight (15%) of 52 eyes had undergone previous vitrectomy; 32 (62%) patients had previous panretinal photocoagulation (PRP) in the affected eye; 16 (31%) had previous anti-VEGF injection for PDR; three (6%) for diabetic macular oedema (DMO); 2 (4%) received anti-VEGF initially indicated for DMO before subsequently requiring injection for PDR; and one received a single injection intraoperatively during vitrectomy. 9 (17%) eyes had no previous diagnosis of PDR and had not received PRP, however, three (6%) had received anti-VEGF for DMO.

Of 48 acute presentations to HES with PDR, 18 (38%) were given anti-VEGF within 72 h and two (4%) had PRP the same day. 16 (33%) were rebooked into the laser clinic, 13 (27%) referred for urgent surgical review, and 17 (35%) advised observation and review in clinic.

Twenty-nine (63%) patients were already under HES care for their retinopathy. 17 (37%) patients had been under DES, of which, 11 had been discharged from HES back to DES: 7 with stable and treated PDR with full PRP; 3 with stable DMO; and 1 patient with early PDR lost to follow-up due to non-attendance. Of these patients discharged from HES, one presented with a TRD, 9 unilateral vitreous haemorrhages (VH) and one bilateral VH. 6 (13%) were under DES for non-referrable retinopathy, of which four were regular non-attenders, and presented to HES during the pandemic with a complication of PDR.

The appointments of the patients who experienced complications were reviewed to establish if their scheduled review appointment was affected due to COVID. Of the 29 patients who were scheduled for HES follow-up, 20 (69%) had their appointment delayed/cancelled due to COVID restrictions.

Ten (59%) of the 17 patients under DES care had attended their most recent scheduled appointment. 9 (53%) had been graded as having non-referrable retinopathy and one (6%) had already been referred to HES for PDR. Two (12%) had their appointment cancelled due to COVID-19 restrictions; and 5 (29%) did not attend their most recent scheduled screening appointment, of which four were regular non-attenders.

Of 52 eyes, there were 6 (12%) new PDR complication presentations to HES, two were screened as having non-referrable retinopathy in DES 7 and 8 months prior to presentation, and 4 were regular non-attenders. Of the new presentations, two presented with NVG and 4 with unilateral vitreous haemorrhage.

Thirty-five (80%) patients who were seen urgently in HES during lockdown were issued a follow-up appointment within the doctor’s recommended timeframe, of which three failed to attend, despite COVID hospital restrictions. Delay in 9 (20%) cases was attributed to capacity issues triggered by COVID. The median time that follow-up appointments were delayed by were 6.5 weeks (IQR 3–11; range 2–22 weeks).

Median follow up duration in this cohort was 6 months (IQR 5–7; range 1–9 months) and 12 eyes (23%) of 11 patients progressed onto vitrectomy. Mean time from presentation to vitrectomy was 16.5 weeks (±12; range 1.5–38). Median vision improved from CF or 2.00 logMAR (IQR 0.4–2.30; range 0.00–2.80) to 0.40 (IQR 0.20–1.00; range − 0.10 – 2.80) at the end of follow-up, *p* = 0.001. There was no statistical difference in visual improvement or speed of improvement in the patients who were managed with IVT initially compared to those who received observation and/or PRP alone (*p* = 0.190) (Table 2).

**Table 2:** BCVA at three timepoints between patients that received anti-VEGF and patients that did not

	IVT ± PRP Median (IQR) [range]	No IVT ± PRP Median (IQR) [range]	***P*** value
**At presentation**	CF (0.70-HM) [0.00–2.80]	1.00 (0.40-HM) [0.00–2.80]	0.140
**At 1 month**	0.60 (0.25-CF) [0.00–2.30]	CF (0.32-HM) [0.00–2.30]	0.186
**At end of study**	0.35 (0.20–0.60) [0.00–2.80]	0.60 (0.15-CF) [− 0.10–2.80]	0.190

*Abbreviations*: *BCVA* best corrected visual acuity, CF counting fingers, *HM* hand movements, *IQR* interquartile range, *IVT* intravitreal injection, *PRP* panretinal photocoagulation

There was no correlation observed between socioeconomic status (SES) and presenting vision, r(49) = − 0.11, *p* = 0.46; and no correlation between SES and final vision at the latest follow-up, r(41)= − 0.02, *p* = 0.89.

## Discussion

To the author’s knowledge this is the first cross-sectional review of one centre’s acute proliferative diabetic retinopathy related complications during the first COVID-19 wave. As routine eye appointments in NHS Grampian were suspended, all patients under HES care were risk stratified in order to identify those at highest risk of sight loss. These exceptional circumstances presented a unique opportunity for a complete dataset as all referrals were received at one point of entry. However, this cohort is likely to underrepresent the true number of patients who suffered a PDR complication as many may have not presented to acute services, particularly those who were shielding or patients who have experienced vitreous haemorrhages in the past.

Although many patients were not attending for their urgent appointments across the UK [19], we observed only three patients who did not attend for their review appointment following initial acute presentation. The reason for non-attendances is open to conjecture and may be due to COVID-19 related illness, fear of exposure to COVID-19 or visual acuity may have improved following initial assessment. It is recognised that patients with diabetes are often overburdened with medical appointments for multiple systemic issues and may miss appointments due to work and family commitments. COVID-19 is unlikely to have had an impact on the four patients who regularly did not attend.

Sixty-three percent of patients who presented were under HES care and two thirds of these had their scheduled review appointment affected by COVID-19 cancellations. We are unable to say whether the complication could have been prevented if their follow-up had not been postponed.

Two thousand six marked the rollout of Scotland’s national screening programme and has been shown to be an effective means of identifying diabetics at high risk of developing sight threatening retinopathy [20]. With the pause of DRS many patients were vulnerable to the effects of PDR. We observed two patients who had their DRS appointment cancelled and subsequently presented with PDR related vitreous haemorrhages. In a report by Forster et al. it was observed that missing one year of screening did not increase the risk detecting referrable retinopathy. However, not attending on two consecutive years increased the odds by 10.84 times of detecting referable retinopathy [21].

Development of an acute complication was seen in patients despite stable appearances at most recent hospital review and screening. Complications were observed in 9 patients who did not have their HES appointment delayed and 9 patients who were identified as having non-referrable retinopathy at screening. The mean time from screening to complication was 8.2 months. Reasons for progression may be the result of more erratic glycaemic control during lockdown, but may also be due to the unpredictability of DR.

Eleven (24%) patients had been discharged from HES back to DRS, 7 of whom had been shown to have stable retinopathy after treatment with PRP. Life-long follow-up is needed for patients with PDR as complications may still occur months to many years after a period of quiescence. The risk of recurrence can be anticipated by previous duration to achieve new vessel regression, duration of diabetes and metabolic control [22]. Despite efforts to optimise modifiable risk factors retinopathy can still progress. Although this poses the question, how safe is it to discharge treated patients with PDR, a good liaison with community optometry, DRS and patient education may allow follow-up of stable treated PDR out with HES, thereby relieving stress on the already stretched HES.

HES have access to wide field fundus imaging, whereas, DRS use two 45-degree field images. Negretti et al. observed 108 eyes and found that 17% of NVE were outside of the DRS imaging fields and 11% of patients with active PDR would have been missed [23]. However, despite DRS not having 100% sensitivity it is considered a safe and effective screening tool to detect PDR. As the referral rate of PDR to ophthalmology in Scotland is reported to be 2.1–2.8% per year for type 1 diabetics and 0.4–0.7% for type 2 [20], the discharge of stable patients avoids over burdening HES resources.

It is well recognised that the progression of diabetic retinopathy is directly related to poor glycaemic control. Our cohort had a mean HbA1c of 78 mmol/l. Lockdown related lifestyle disruption appears to have had an impact on glycaemic control. Khare et al. demonstrated a 0.51% HbA1c rise in an Indian population over a 68 day lockdown [24]. The compliance with diabetic medication and healthy living habits was significantly reduced after lockdown [25]. Fernandez et al. observed different results where type 1 diabetics in Spain using flash glucose monitoring system recorded improved glycaemic control over the period of lockdown [26].

One tertiary centre in Greece described the negative effect on visual acuity and progression to active PDR as a result of deferring appointments during lockdown [15]. Ghosal et al. created a predictive model with regard to diabetic glycaemic control during lockdown using multivariate regression analysis [27]. They predicted an HbA1c increase of 3.68% over 45 days and a 2.9% increase in ocular PDR complication rates.

Although it is documented in the literature that deprivation is a major determinant of health and in the case of diabetic retinopathy, significant loss of vision, we found no correlation between SES and severity of vision loss at presentation or final visual outcome at last follow-up. Denniston et al. reports an association between deprivation and late disease presentation with significant vision loss [28]. In our cohort, the five non-attenders to screening were from more privileged areas. This may be attributed to the general demographic of Grampian’s population being more affluent and all patients out with Aberdeen City are equally disadvantaged by geography as great distances must be travelled in order to seek specialist ophthalmic support. It may also suggest that all patients were affected by COVID-19 regardless of SES.

Twenty-three eyes (44%) of 18 patients received anti-VEGF injection as part of initial management of their PDR complication. We observed no statistical difference in visual improvement nor speed of visual improvement between the eyes that were injected and those that did not. Although all patients were offered follow-up, some patients received more regular follow-up than others due to complexity. In order to reduce omissions in data, visual acuities were taken at presentation, at 1 month and at 6 months.

The evidence for the use of intravitreal anti-VEGF injection in the context of VH secondary to PDR remains unclear and although there appear to be visual benefits, they are not long lasting. DRCR net conducted a randomised controlled trial where participants with VH secondary to PDR received either ranibizumab or saline [29]. Little significance in vitrectomy rates were observed between the groups, although, patients who received anti-VEGF had greater improvement in visual acuity and reduced rate of VH recurrence in the short term. Huang et al. suggested vitreous haemorrhage resolution hastened with bevacizumab to a mean of 12 weeks compared to controls at 18 weeks [30].

Of 850 patients diagnosed with PDR in a Finnish population, Wirkkala et al. observed vitreous haemorrhage occur in 16% of type 1 diabetics and 9% type 2 diabetics [31]. Two thirds received anti-VEGF injection and VH clearance occurred within 3 months in 92% of these eyes. They concluded that timely injection accelerated VH resolution, improved visual outcome, reduced recurrent VHs and reduced the need for vitrectomy by 72% over the 5 year study period.

The previously mentioned studies included patients with variable levels of PRP. Park et al. studied the effect of bevacizumab on diabetic VH in patients with complete PRP [32]. Injection increased the likelihood of VH clearance and reduced the need for vitrectomy. Although a greater visual improvement was seen, this effect was also short lived.

Urgent vitrectomy offers a surgical option for the rapid clearance of an acute diabetic VH. Although due to the invasive nature, associated risks and resource dependence other treatment modalities for the initial management of diabetic VH have been investigated. Protocol AB identified no significant difference in visual outcome at 6 months for patients initially treated with aflibercept compared to urgent vitrectomy with panretinal photocoagulation [33]. Surgery was subsequently avoided in two thirds of patients that received aflibercept.

A concern when administering anti-VEGF to patients with advanced PDR is the risk of tractional detachment. Although there is strong evidence for anti-VEGF’s role in the management of PDR, it can facilitate fibrosis and subsequent traction through upregulation of the fibrin-fibronectin complex [34]. Of 608 eyes that received anti-VEGF prior to vitrectomy for active PDR and non-macular involving traction, the incidence of tractional macula detachment was 10% at the time of surgery. The risk of detachment increased significantly if the surgery was performed greater than 6 days after injection.

With the concern of COVID-19 virus transmission via direct contact, droplet and airborne routes, efforts were made to ensure the time in close proximity with patients was kept to a minimum. Despite these risk mitigation measures, the delivery of urgent PRP was not delayed in this cohort of patients. In those previously treated with PRP, anti-VEGF was a practical option to manage active PDR due to the short procedural time.

Anti-VEGF therapy is a useful adjunct to PRP in the management of PDR [35], particularly if PRP is not practical. Protocol S suggested that ranibizumab is non-inferior and may be more effective than PRP for visual acuity at 2 years, although half of the participants in the PRP group also received anti-VEGF treatment for DMO [36]. Indefinite intraocular injections is invasive, disruptive and costly when the alternative can be completed in a few sessions and without the risk of endophthalmitis or retinal detachment.

The CLARITY study demonstrated the advantageous effect on visual outcome at one year with a lower incidence of vitreous haemorrhage following loading treatment with aflibercept for PDR over standard PRP treatment [7]. A meta-analysis by Gao et al. demonstrated fewer PDR complications with the use of anti-VEGF when compared to PRP alone [37]. Through the use of OCTA (optical coherence tomography angiography), He et al. suggested that combination PRP plus anti-VEGF treatment was more effective at regressing NVE than PRP alone [38]. If combination treatment were to be offered in routine practise one must consider the practicality in an era of COVID-19 risk mitigation measures for example, cost, availability of resources and multiple hospital visits. PRP is thought to remain the mainstay in management in reducing the risk of sight threatening diabetic retinopathy.

The need for vitrectomy and development of neovascular glaucoma are advanced complications of PDR. We observed 11 eyes of 12 patients progress onto vitrectomy and 3 eyes developed neovascular glaucoma: one responded well to both IVT and PRP, and two required further IOP lowering procedures. The ETDRS study reported the 5-year incidence rate of vitrectomy to be 5.3% in diabetics with established DR [39]. The progression to vitrectomy has reduced over the past three decades. In 2010, Ostri et al. reports a 10-year incidence vitrectomy rate of 2.9% in type 1 diabetics [40].

In a Northern English population, Vaideanu et al. report that although the prevalence of diabetes had increased from 2.8 to 5.5% from 2000 to 2010, respectively, the rate of PDR within the diabetic population had reduced from 2.4 to 1.8% and the vitrectomy rate in these patients had reduced from 7.7 to 5.7% over the same time period [41]. This can be partly attributable to the introduction of screening, allowing patients to receive timely treatment prior to the development of sight threatening DR.

In our cohort, with 6 months follow-up, we observed a 32% re-bleed rate (*n* = 15) and a 25% (*n* = 3) rate of post vitrectomy vitreous haemorrhage. At 6 months post vitrectomy, 50% (*n* = 6) had a BCVA better than 6/60 and 50% had CF vision or worse. Yorston et al. evaluated the post-operative outcomes of patients who underwent vitrectomy for PDR in a Scottish population [42]. Twenty-two percent re-bled within 6 months, 3% re-detached and 3% progressed onto neovascular glaucoma. The visual outcomes were described as unpredictable with 72% achieving a vision of 6/60 or better and 16% had CF vision or worse.

Limitations of the study include a lack of control group to compare how presentations to HES have changed. Comparing our data to pre-COVID-19 conditions would have been preferred to determine if the differences in how patients had presented were significant. As a result of Grampian’s geography and the strong relationship between community and hospital eye care asynchronous telemedicine is commonplace, allowing many patients to be managed in the community. Comparison with prospective “post-COVID-19” data collection will be the focus of future research. Modifiable risk factors that affect diabetic retinopathy including hypertension and BMI were not included.

As patients with advanced complications of PDR are, by definition, complex there is no clear consensus on optimal management. Management styles are likely to have been influenced by social distancing measures, for example, intravitreal injection may have been preferentially offered over PRP. This decision-making process will have inter-clinician variation within the unit.

Seven eyes of 6 patients had no follow-up data and efforts were made to contact patients and optometry practices to organise review. Closer review of the patients that were under DRS care would be of interest to determine if the retinal photograph and outcome of the most recent appointment demonstrated evidence of evolving PDR activity.

## Conclusion

Although routine diabetic screening and HES review was paused, patients who suffered acute diabetic complications were assessed in one of six emergency optometry services. Aided by tele-ophthalmology, referrals to HES were prioritised by urgency. Despite widespread cancellations 80% of follow-up appointments were met in a timely fashion. Retinal laser, IVT and surgical intervention were adapted to ensure the COVID-19 risk was reduced for both patient and physician to ensure the continued delivery of high-quality care despite social distancing measures.

## Data Availability

The datasets used and analysed during the current study are available from the corresponding author on reasonable request.
